# Alteration of the Risk of Oral Pre-Cancer and Cancer in North India Population by *CYP1A1* Polymorphism Genotypes and Haplotype

**DOI:** 10.31557/APJCP.2019.20.2.345

**Published:** 2019

**Authors:** Kumud Nigam, Somali Sanyal, Shalini Gupta, Om Prakash Gupta, Abbas Ali Mahdi, M L B Bhatt

**Affiliations:** 1 *Department of Oral Pathology and Microbiology,*; 4 *Department of Biochemistry, *; 3 *5Department of Radiotherapy, King George’s Medical University, *; 2 *Amity Institute of Biotechnology, Amity University, Lucknow, India, *; 3 *Department of Surgery, SHM Medical College, Saharanpur, U.P. *

**Keywords:** Oral pre cancer and cancer, CYP1A1, PCR, RFLP, gene polymorphism

## Abstract

**Background:**

The aim of this study was to evaluate any association between CYP1A1 (T6235C and C4887A, A4889G) gene polymorphisms and the risk of oral pre-cancer and cancer.

**Methods:**

In the present study, 250 patients with oral pre-cancer and/or cancer and 250 healthy controls were genotyped for CYP1A1 T6235C, C4887A and A4889G polymorphisms by the PCR-RFLP method.

**Results:**

None of the CYP1A1 polymorphisms were associated with the risk of either oral cancer or pre cancer. Nor were any links with clinical parameters of oral cancer found. However, among the consumers of areca nut/pan masala the TC, CA and AG genotypes respectively for the CYP1A1 T6235C,C4887Aand A4889G polymorphisms were significantly more frequent in controls compared to cases (p values for cases vs. controls of 0.0032, 0.0019 and 0.0009, respectively). Similarly, compared to the haplotype TCA, TAG constituted by CYP1A1 T6235C and C4887A and A4889G was more common in controls (6.88%) than in cases (4.07%).

**Conclusion:**

Our results suggest that genotypes regarding CYP1A1 polymorphisms may modulate the risk of oral cancer and pre-cancer among the areca nut/pan masala consumers. The haplotype may also exert an influence in our north Indian population.

## Introduction

Oral cancer represents an important problem worldwide. The incidence and prevalence rates for these tumors are double in men than in women. Cancers of the oral cavity rank as the eighth most common cancer among men, being responsible for 3% of the cancers diagnosed in this gender (Ferlay et al., 2002). Mortality rates are substantially lower thanincidence rates. According to theWorld Health Organization(WHO) data, the standardized mortality rate for 2002 was 2.2 deaths per 100,000 population. Invasive oral squamous cell carcinoma is often preceded by the presence of clinically identifiable dysplasia of the oral mucosa (Neville and Day, 2002). These include: erythroplakia, non-homogeneous leukoplakia, erosive lichen planus, oral submucous fibrosis and actinic keratosis (Waal, 2009; Warnakulasuriya et al., 2007). This precancerous lesion is a morphologically altered tissue in which cancer is more likely to occur than in its apparently normal counterpart. 

**Table 1:** Primers and Restriction Enzymes used for Genotyping Various Polymorphisms in Oral Pre Cancer and Cancer Patients and Controls

Gene	Primer sequence	Annealing temp. (ºC)	Restriction enzyme
CYPIAI(−6235) T/C	F CAG TGA AGA GGT GTA GCC GCT	62	MspI
	R TAG GAG TCT TGT CTC ATG CCT		
CYP1A1(-4887) C/A	F TGG GCA AGC GGA AGTGT	60	MboII
	R CA GGA AGA GAA AGA CC		
CYP1A1(-4889) A/G	F GCTTGCCTGTCCTCTATC	60	NcoI
	R AAAGACCTCCCAGCGGGTAA		

**Table 2:** Demographic and Risk factors in Patient and Controls and Their Association with Risk of Oral Pre Cancer and Cancer

Demographic character	Cases(n=250) (%)	Control (n=250) (%)	P- value
Male	150 (60)	175 (70)	0.3131
Female	100 (40)	75 (30)	0.1236
Age distribution			
<20 – 40	160 (64.00)	131 (52.40)	0.2010
<40- 70	90 (36.00)	119 (47.60)	0.1088
Habitual risk			
Areca nut/ Pan masala	80 (32)	165 (66)	0.0011*
Alcohol consumption	40 (16)	10 (4)	0.0011*
Smoking	60 (24)	30 (12)	0.0051*
Tobacco chewing	70 (28)	45 (18)	0.0455*
Type of cancer			
Leukoplakia	50 (20.00)	-	-
O.S.M.F	100 (40.00)	-	-
Lichen Planus	50 (20.00)	-	-
Malignancy	50 (20.00)	-	-
TNM staging of oral cancer(Malignancy) -			
Tumor Stage		-	-
I	18 (36.00)		
II	16 (32.00)		
III	10 (20.00)		
IV	06 (12.00)		
Tumor T Status - -			
≤T2	31 (62.00)		
>T2	19 (38.00)		
Lymph Node - -			
N0	37 (74.00)		
N1+N2	13 (26.00)		
Metastasis - -			
M0	29 (58.00)		
M1	21 (42.00)		
Cell differentiated grade - -			
Grade 1	26 (52.00)		
>Grade 1	24 (48.00)		

*, significant value

**Table 3:** Allelic Distribution of CYP1A1 (T6235C), CYP1A1 (C4887A), CYP1A1 (A4889G) Gene in Oral Pre Cancer and Cancer and Controls

	Cases(n=250)(%)	Controls(n=250)(%)	Odds Ratio	95% CI
CYP1A1 (T6235C) Genotypes				
TT	104 (41.60)	106 (42.40)	Reference	Reference
TC	110 (44.00)	112 (44.80)	1.001	0.6863-1.460
CC	36 (14.40)	32 (12.80)	1.147	0.663-1.98
T	319 (63.80)	324 (64.80)	-	-
C	181 (36.20)	176 (35.20)	1.045	0.806-1.353
CYP1A1 (C4887A) Genotypes				
CC	127 (50.80)	139 (55.60)	Reference	Reference
CA	99 (39.60)	92 (36.80)	1.178	0.811-1.709
AA	24 (9.60)	19 (7.60)	1.383	0.722-2.644
C	353 (70.60)	370 (74.00)	-	-
A	147 (29.40)	130 (26.00)	1.185	0.898-1.564
CYP1A1 (A4889G) Genotypes				
AA	177 (70.80)	192 (76.80)	Reference	Reference
AG	63 (25.20)	55 (22.00)	1.243	0.820-1.883
GG	10 (4.00)	03 (1.20)	3.616	0.978-13.35
A	417 (83.40)	439 (87.80)	-	-
G	83 (16.60)	61 (12.20)	1.432	1.003-2.047

After adjusting age and sex the GG genotype for CYP1A1 (A4889G) polymorphism found to be associated with significantly reduce risk for development of oral cancer and oral pre cancer. ( odd ratio 0.24, 95% CI 0.06-0.91 , p value 0.025.

**Table 4:** Analysis of CYP1A1 (T6235C), CYP1A1 (C4887A), CYP1A1 (A4889G) Gene Polymorphism in Oral Pre Cancer and Cancer and Controls with Individuals under Smoking, Masala Tobacco Chewing and Alcohol Consumption Criteria

Genotypes	Cases	Controls	Odds Ratio			95% CI
Smoking	(n=60)(%)	(n=30)(%)				
CYP1A1 (T6235C) genotypes						
TT	32 (53.33)	19 (63.33)	Reference			Reference
TC	28 (46.67)	08 (26.67)	2.078			0.788-5.480
CC	00 (00.00)	03 (10.00)	-			-
T	92 (76.67)	46 (76.67)	-			-
C	28 (23.33)	14 (23.33)	1.000			0.4805-2.081
CYP1A1 (C4887A) genotypes						
CC	28 (46.67)	19 (63.34)	Reference			Reference
CA	31 (51.67)	11 (36.66)	1.912			0.776-4.711
AA	01 (1.66)	00 (00.00)	-			-
C	73 (60.83)	36 (60.00)	-			-
A	47 (39.16)	24 (40.00)	0.965			0.512-1.820
CYP1A1 (A4889G) genotypes						
AA	29 (48.33)	18 (60.00)	Reference			Reference
AG	27 (45.00)	10 (33.33)	1.676			0.658-4.265
GG	04 (6.66)	02 (6.66)	-			-
A	85 (70.83)	46 (76.66)	-			-
G	35 (29.16)	14 (23.33)	1.353			0.661-2.769
Masala, Tobacco Chewing	(n=70)	(n=45)				
CYP1A1 (T6235C) genotypes						
TT	41 (58.57)	26 (57.78)	Reference			Reference
TC	28 (40.00)	17 (37.78)	1.044			0.479-2.273
CC	01 (1.43)	02 (4.44)	0.317			0.027-3.67
T	110 (78.57)	69 (76.67)	-			-
C	30 (21.43)	21 (23.33)	0.896			0.475-1.689
CYP1A1 (C4887A) genotypes						
CC	25 (35.72)	24 (53.34)	Reference			Reference
CA	43 (61.42)	20 (44.44)	2.064			0.9541-4.465
AA	02 (2.86)	01 (2.22)	1.920			0.163-22.59
C	84 (60.00)	57 (63.34)	-			-
A	56 (40.00)	33 (36.66)	1.152			0.666-1.988
CYP1A1 (A4889G) genotypes						
AA	26 (37.14)	23 (25.55)	Reference			Reference
AG	42 (60.00)	19 (21.11)	1.955			0.896-4.267
GG	02 (2.85)	03 (3.33)	-			-
A	94 (67.14)	65 (72.22)	-			-
G	46 (32.85)	25 (27.77)	1.272			0.711-2.274
Alcohol Consumption	(n=40)	(n=10)				
CYP1A1 (T6235C) genotypes						
TT	26 (65.00)	06 (60.00)	Reference			Reference
TC	14 (35.00)	04 (40.00)	0.807			0.194-3.350
CC	00 (00.00)	00 (00.00)	-			-
T	66 (82.50)	16 (80.00)	-			-
C	14 (17.50)	04 (20.00)	0.848			0.245-2.928
CYP1A1 (C4887A) genotypes						
CC	17 (42.50)	06 (60.00)		Reference	Reference	
CA	22 (55.00)	04 (40.00)		1.941	0.471-7.99	
AA	01 (2.50)	00 (00.00)		-	-	
C	46 (57.50)	11 (55.00)		-	-	
A	34 (42.50)	09 (45.00)		0.903	0.336-2.423	
CYP1A1 (A4889G)genotypes						
AA	18 (45.00)	06 (60.00)		Reference	Reference	
AG	22 (55.00)	04 (40.00)		1.833	0.447-7.513	
GG	00 (00.00)	00 (00.00)		-	-	
A	58 (72.50)	16 (80.00)		-	-	
G	22 (27.50)	04 (20.00)		1.517	0.456-5.042	
Areca nut/Pan masala	(n=80)	(n=165)				
CYP1A1 (T6235C)genotypes						
TT	41 (51.25)	54 (32.72)		Reference	Reference	
TC	26 (32.50)	87 (52.73)		0.393	0.216-0.715	
CC	13 (16.25)	24 (14.55)		-	-	
T	108 (67.50)	195 (59.10)		-	-	
C	52 (32.50)	135 (40.90)		0.695	0.467-1.035	
CYP1A1 (C4887A)genotypes						
CC	39 (48.75)	49 (29.69)		Reference	Reference	
CA	27 (33.75)	91 (55.15)		0.372	0.204-0.680	
AA	14 (17.50)	25 (15.15)		0.703	0.323-1.532	
C	105 (65.62)	189 (57.27)		-	-	
A	55 (34.37)	141 (42.72)		0.7021	0.4742-1.040	
CYP1A1 (A4889G)genotypes						
AA	43 (53.75)	55 (33.33)		Reference	Reference	
AG	25 (31.25)	90 (54.54)		0.3553	0.1957-0.6451	
GG	12 (15.00)	20 (12.12)		0.7674	0.3381-1.742	
A	111 (69.37)	200 (60.60)				
G	49 (30.62)	130 (39.39)		0.6791	0.4542-1.015	

*, significant value

**Table 5:** Analysis of Genotype and Allele Frequencies in CYP1A1 (T6235C), CYP1A1 (C4887A), CYP1A1 (A4889G) Gene Polymorphism with Tumor Stage, Tumor T Status, Lymph Node, Metastasis and Cell Differentiated Grade in Oral pre Cancer and Cancer

CYP1A1 (T6235C), CYP1A1 (C4887A), CYP1A1 (A4889G) Genotypes/Alleles							
Tumor Stage	I+II (n=34)	III+IV (n=16)	P-value		Odds Ratio		95% CI
CYP1A1 (T6235C)							
TT	19 (55.88%)	06 (37.50%)	Reference		Reference		Reference
TC	14 (41.18%)	09 (56.25%)	0.4133		0.4912		0.1418-1.702
CC	01 (2.94%)	01 (6.25%)	-		-		-
T	52 (76.47%)	21 (65.62%)	-		-		-
C	16 (23.52%)	11 (34.37%)	0.3691		0.5874		0.2341-1.474
CYP1A1 (C4887A)							
CC	16 (47.05%)	08 (50.00%)	Reference		Reference		Reference
CA	15 (44.11%)	06 (37.50%)	0.9828		1.25		0.3504-4.459
AA	03 (8.80%)	02 (12.50%)	0.7754		0.75		0.1035-5.436
C	47 (69.11%)	22 (68.75%)	-		-		-
A	21 (30.88%)	10 (31.25%)	0.9704		0.983		0.3966-2.436
CYP1A1 (A4889G)							
AA	11 (32.35%)	08 (50.00%)	Reference		Reference		Reference
AG	21 (61.76%)	05 (31.25%)	0.1805		3.055		0.8040-11.60
GG	02 (5.88%)	03 (18.75%)	0.8335		0.4848		0.650-3.612
A	43 (63.23%)	21 (65.62%)	-		-		-
G	25 (36.76%)	11 (34.37%)	0.9929		1.11		0.4601-2.678
Tumor T Status	< T2 (n=31)	> T2 (n=19)					
CYP1A1 (T6235C)							
TT	12 (38.70%)	05 (26.31%)	Reference		Reference		Reference
TC	10 (32.25%)	05 (26.31%)	0.8112		0.8333		0.1864-3.725
CC	09 (29.03%)	09 (47.36%)	0.3695		0.4167		0.1034-1.679
T	34 (54.83%)	15 (39.47%)	-		-		-
C	28 (45.16%)	23 (60.52%)	0.1985		0.5371		0.2364-1.220
CYP1A1 (C4887A)							
CC	21 (67.74%)	09 (47.36%)	Reference		Reference		Reference
CA	09 (29.03%)	08 (42.10%)	0.3933		0.4821		0.1407-1.653
AA	01 (3.22%)	02 (10.52%)	-		-		-
C	51 (82.25%)	26 (68.42%)	-		-		-
A	11 (17.74%)	12 (31.57%)	0.1766		0.4673		0.1817-1.202
CYP1A1 (A4889G)							
AA	19 (61.29%)	11 (57.89%)	Reference		Reference		Reference
AG	10 (32.25%)	05 (26.31%)	0.8257		1.158		0.3138-4.273
GG	02 (6.46%)	03 (15.78%)	0.622		0.386		0.556-2.680
A	48 (77.42%)	27 (71.05%)	-		-		-
G	14 (22.58%)	11 (28.94%)	0.6342		0.7159		0.2853-1.796
Lymph Node	N0 (n=37)	N1+N2 (n=13)					
CYP1A1 (T6235C)							
TT	29 (78.37%)	07 (53.84%0	Reference		Reference		Reference
TC	08 (21.63%)	05 (38.47%)	0.3219		0.3862		0.096-1.550
CC	00 (00.00%)	01 (7.69%)	-		-		-
T	66 (89.18%)	19 (73.07%)	-		-		-
CYP1A1 (C4887A)							
CC	18 (48.64%)	06 (46.15%)	Reference	Reference		Reference	
CA	17 (45.94%)	05 (38.46%)	0.8567	1.133		0.291-4.414	
AA	02 (5.42%)	02 (15.38%)	0.6694	0.333		0.038-2.912	
C	53 (71.62%)	17 (65.38%)	-	-		-	
A	21 (28.38%)	09 (34.61%)	0.7277	0.7484		0.2885-1.941	
CYP1A1 (A4889G)							
AA	21 (56.75%)	08 (61.53%)	Reference	Reference		Reference	
AG	13 (35.13%)	03 (23.07%)	0.7658	1.651		0.369-7.374	
GG	03 (8.10%)	02 (15.38%)	0.9751	0.5714		0.799-4.082	
A	55 (74.33%)	19 (73.07%)	-	-		-	
G	19 (25.67%)	07 (26.92%)	0.9007	0.937		0.340-2.579	
Metastasis	M0 (n=29)	M1 (n=21)					
CYP1A1 (A4889G)							
AA	22 (75.86%)	17 (80.95%)	Reference	Reference		Reference	
AG	06 (20.68%)	04 (19.04%)	0.8378	1.159		0.281-4.771	
GG	01 (3.44%)	00 (00.00%)	-	-		-	
A	50 (86.20%)	38 (90.47%)	-	-		-	
G	08 (13.79%)	04 (9.52%)	0.7364	1.52		0.425-5.426	
CYP1A1 (C4887A)							
CC	16 (55.17%)	11 (52.38%)	Reference	Reference		Reference	
CA	11 (37.93%)	09 (42.85%)	0.7703	0.84		0.261-2.704	
AA	02 (6.89%)	01 (4.76%)	0.8038	1.375		0.110-17.10	
C	43 (74.13%)	31 (73.80%)	-	-		-	
A	15 (25.86%)	11 (26.19%)	0.9705	0.983		0.397-2.430	
CYP1A1 (A4889G)							
AA	19 (65.51%)	13 (61.90%)	Reference	Reference		Reference	
AG	07 (24.13%)	05 (23.80%)	0.9501	0.957		0.249-3.686	
GG	03 (10.34%)	03 (14.28%)	0.6695	0.6842		0.119-3.935	
A	45 (77.58%)	31 (73.80%)	-	-		-	
G	13 (22.41%)	11 (26.19%)	0.8421	0.8141		0.323-2.052	
Cell differentiated grade	Grade 1 (n=26)	> Grade 1 (n=24)					
CYP1A1 (T6235C)							
GG	16 (61.53%)	16 (66.66%)	Reference	Reference		Reference	
GA	09 (34.61%)	07 (29.16%)	0.9186	1.286		0.384-4.298	
AA	01 (3.84%)	01 (4.16%)	-	-		-	
G	41 (78.84%)	39 (81.25%)	-	-		-	
A	11 (21.15%)	09 (18.85%)	0.9601	1.163		0.434-3.111	
CYP1A1 (T6235C)							
AA	13 (50.00%)	14 (58.33%)	Reference	Reference		Reference	
AG	11 (42.30%)	09 (37.50%)	0.8654	1.316		0.412-4.201	
GG	02 (7.69%)	01 (4.16%)	-	-		-	
A	37 (71.15%)	37 (77.08%)	-	-		-	
G	15 (28.84%)	11 (22.91%)	0.6547	1.364		0.5534-3.360	
CYP1A1 (A4889G)							
AA	19 (73.07%)	15 (62.50%)	Reference	Reference		Reference	
AG	05 (19.23%)	09 (37.50%)	0.3408	0.438		0.121-1.587	
GG	02 (7.69%)	00 (00.00%)	0.6228	3.974		0.177-89.06	
A	43 (82.69%)	39 (81.25%)	-	-		-	
G	09 (17.30%)	09 (18.75%)	0.8512	0.907		0.326-2.517	

*, significant value

**Table 6:** Distribution of Different Haplotype of CYP1A1 (T6235C), CYP1A1 (C4887A), CYP1A1 (A4889G) Polymorphism in Oral Pre Cancer and Cancer and Controls

Haplotype	Cases (n=250) (%)	Controls (n=250) (%)	Odds Ratio	95% CI
TCA	51.76	52.25	1	-
CCA	18.84	21.75	1.22	0.81-1.85
CAG	9.72	8.13	0.65	0.36-1.17
TAA	4.96	8.49	1.55	0.76-3.17
CAA	6.59	7.84	0.62	0.33-1.19
TAG	5.39	6.88	0.48	0.24-0.98
CCG	0.00	0.00	-	-

**Figure 1:**
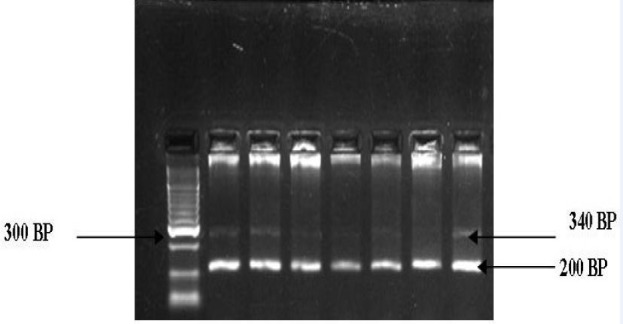
3% Agarose Gel Analysis of CYP1A1 (T6235C) polymorphism. Lane 1 100bp Ladder, Lane 2,3,4,5,6,7 340bp,200bp

**Figure 2:**
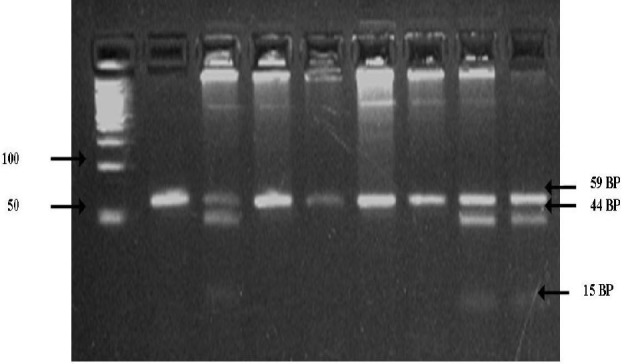
24% Agarose gel analysis of CYP1A1 (C4887A) polymorphism. Lane 1 100bp Ladder, Lane 2,4,5,6,7,8 CC 59bp,lane 3,9,10 CA 59,44,15

**Figure 3:**
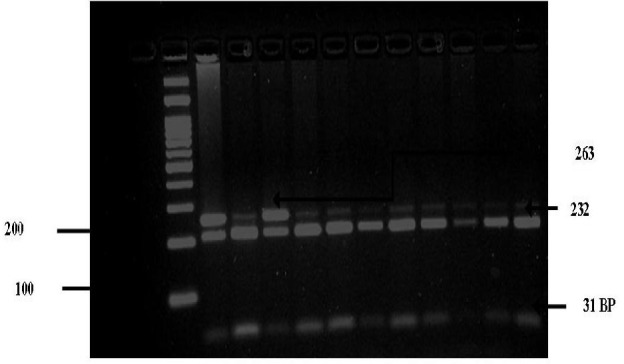
3% Agarose gel analysis of CYP1A1 (A4889G) polymorphism. Lane 1 100bp Ladder, Lane 2,4 AG genotype 263,233,31 bp, Lane 5,6,7,8,9,10 GG genotype 232,31 bp

Tobacco and alcohol usage are strong risk factors and have been causally associated with the development of oral cancer (Petersen, 2009). In India, tobacco exposure occurs in complex patterns due to the consumption of a wide range of smoked and smokeless tobacco products. Apart from this, molecular epidemiological studies have provided concrete evidence that genetic predisposition play an important role in the aetiology of this malignancy (Gatt_as et al., 2009).

Enzymes like CYP1A1 are generally responsible for biotransformation of several tobacco-related pro-carcinogens such as polycyclic aromatic hydrocarbons (PAHs), nitrosamines and aromatic amines (Sikdar et al., 2003) into reactive electrophilic intermediate metabolites that can damage DNA. Among the different reactions catalyzed by CYP1A1, hydroxylation at a vacant position of an aromatic ring is considered to be the hallmark for the initiation of carcinogenesis, through the formation of highly reactive conversion products that can cause oncogenic mutations in experimental animals and humans (Wei et al., 1996; Buterin et al., 2000) The CYP1A1 gene is located on chromosome 15q22-24 and has seven exons (Hildebrand et al., 1985). Three polymorphisms had been described in the human CYP1A1 gene: a Msp I RFLP in the 3’ UTR (T6235C, rs4646903), an adenine to guanine transition in the heme-binding domain of exon 7 (A4889G, rs1048943) which causes Ile .Val exchange in residue 462 and an African-American-specific RFLP in exon 7 (C4887A) causes an Thr.Asn exchange. These genetic variants of CYP1A1 genes, besides causing enhanced enzyme activity, are also known to play a major role in the pathogenesis of several cancers (Singh et al., 2007; Wright et al., 2010; Kristiansen et al., 2011; Singh et al., 2007; Wright et al., 2010; Kristensen et al., 2011).

Although a largebody of experimental results points towards a positiveassociation of CYP1A1 genetic polymorphisms and canceroccurrence, further investigation is required for such findingsto be extrapolated successfully to human populations.The present study aimed to find any association between CYP1A1 T6235C,C4887Aand A4889G polymorphisms with the risk of oral cancer and pre cancer.

## Materials and Methods

*Study Subjects*

The study was undertaken on a total of 250 patients with previously treated and pathologically confirmed oral pre cancer and cancer who were registered at department of Oral Pathology and Microbiology, King George’s Medical University and 250 healthy controls.Oral cancer sample were staged according to TNM staging system. Variables included for example age, gender, smoking, tobacco and pan masala chewing status of the study subjects are detailed in Table 2. This study was approved by the Institutional Ethics Committee of the King George’s Medical University, Lucknow. An informed written consent was obtained from all subjects. Ethical clearance was obtained from institutional ethical committee. Venous blood samples were collected in EDTA tubes and stored at −80 °C, till DNA extraction. Genomic DNA extraction from blood samples was carried out by salting out method.

*Genotyping by RFLP*

Genotyping for polymorphisms in CYP1A1 (6235) T/C, CYP1A1 (4887) C/A, CYP1A1 (4889) A/G were detected using PCR--RFLP technique.PCR products were generated by using 10 ng of genomic DNA in 10 ml volumereactions containing 20 mMTris--HCl, 50 mMKCl, 2.0 mM MgCl_2_, 0.11 mMeach dNTP, 0.3 mM each primer (Table 1) and 0.3 U Platinum Taq DNApolymerase (Invitrogen, Paisley, UK). PCR products weredigested with the appropriate MspI enzyme to screen for the CYP1A1 (T6235C) polymorphism (Figure 1),MboII enzyme to screen for the CYP1A1 (C4887A) polymorphism (Figure 2) , NcoI enzyme to screen for the CYP1A1 (A4889G) polymorphism (Figure 3) that recognized and cut wild-type, homozygous, heterozygous sequences at 37 ^o^C for at over night. The digested PCR products were resolved on a 2% agarose gel and stained with ethidium bromidefor visualization under UV light. The genotype results were regularlychecked and confirmed by direct DNA sequencing of the amplified fragments. Most of the assays were carried out including samples with knowngenotypes as controls. Genotypes for polymorphisms without previouslyknown samples were identified by sequencing twice independently; such samples were subsequently used as controls.

*Statistical analysis*

The significance of this study was evaluated by chi-square test. Odds ratio (OR) was calculated as an estimate of relative risk of having disease according to the relative frequency of different genotypes among the cases as well as the controls. P value was considered significant at <0.05.SNPstats was employed to construct haplotypes. The extent of linkage disequilibrium (LD) was expressed in the maximum likelihood estimate of disequilibrium, D’

## Results

*Cohort Characteristics*

Present study included 200 oral pre cancer and 50 oral cancer cases; out of those, 150 were males and 100 were females. Among the 250 healthy controls 175 were males and 75 were females. Male and female ratio was similar in both case and control. Distribution of age was also similar among the cases and control as detailed in Table 2. Habit of smoking, tobacco chewing and alcohol drinking are more prevalent in the cases compared to control. In contrast consumption of areca nut/pan masala was more prevalent in the control population than the cases. Other clinical data including, tumor stage, grade, T status, lymph node involvement and metastasis about the oral cancer patients are detailed in Table 2.

*Association of CYP1A1 polymorphisms with the risk of oral cancer and oral pre cancer*

Distribution of different genotypes for CYP1A1 (6235) T/C, (4887) C/A, and (4889) A/G polymorphisms among the cases and controls are detailed in Table 3. None of the genotypes for the above mentioned polymorphism showed significant association with the risk for developing oral diseases (Table 3). However after adjusting the odds ratio for age and sex the GG genotype for CYP1A1 (A4889G) polymorphism found to be associated with significantly reduce risk for development of oral cancer and oral pre cancer (odd ratio 0.24, 95% CI 0.06-0.91 , p value 0.025).

The risk of disease was further evaluated among the smokers, tobacco chewers, alcohol consumers and areca nut/pan masala chewers. Distribution of different genotypes for CYP1A1 (6235) T/C, (4887) C/A, and (4889) A/G polymorphisms among the above mentioned stratified population are detailed in Table 4. Among the smokers, tobacco chewers and alcohol consumers the frequency of different genotypes for the above said polymorphisms were almost similar while the cases and controls were compared. However, the risk of disease were significantly lower with the TC, CA and AG genotypes for the CYP1A1 (6235) T/C, (4887) C/A, and (4889) A/G polymorphisms respectively (Table 4).

*Association of haplotype of CYP1A1 polymorphisms with the risk of oral cancer and oral pre cancer*

Haplotyping of CYP1A1 (6235) T/C, (4887) C/A, and (4889) A/G polymorphisms generated 7 different haplotypes as detailed in Table 5. The frequency of haplotype TAG was significantly lower in cases (4.07%) compared to healthy controls (7%). The association was more pronounced in male (OR=0.31 95% CI 0.12 - 0.79) than in female (OR= 1.11, 95% CI 0.37 - 3.33). The frequencies of other haplotypes were almost similar between cases and controls. Further, the above polymorphisms of eNOS were not in significant LD asD’ = 0.33 for (6235) T/C and (4887) C/A, 0.430 for (6235) T/C and (4889) A/G and 0.99 for (4887) C/A, and (4889) A/G.

*Association of CYP1A1 polymorphisms with the clinical parameters of oral cancer*

Genotypes from different CYP1A1 polymorphisms were evaluated for their association with different clinical parameters of oral cancer including tumour stage, grade, T status, lymph node involvement and metastasis. None of genotype showed any association with the above said parameters as detailed in Table 6.

## Discussion

The present observational study was designed to examine theimpact of CYP1A1T6235C,C4887Aand A4889G polymorphismson oral oncogenesis. The study was done by comparing the genotypes of oral pre cancer and cancer patients with healthy controls matched for age, gender, and other habitual risk. We have observed that the haplotypes of CYP1A1 polymorphisms influenced the risk of oral cancer and pre cancer in the population. We have alsodocumented an interaction of genotypes of CYP1A1 polymorphisms with the consumption of areca nut and pan masala as certain genotypes from CYP1A1T6235C, C4887A and A4889G polymorphisms found to alter risk of oral cancer and pre cancer in this group.

Cytochrome P450 1A1 (CYP1A1) is a key enzyme in phase I bioactivation (Nebert et al., 1996), which activates procarcinogens to genotoxic electrophilic intermediates. It contributes to aryl hydrocarbon hydroxylase activity, catalyzing the first step in the metabolism of a number of polycyclic aromatic hydrocarbons (PAHs). It is also involved in estrogen metabolism, catalyzing the hydroxylation of 17β-estradiol at the C-2 position (Masson et al., 2005). 

CYP1A1 polymorphisms have been extensively studied with regard to oral cancer risk. Although some studies report increased risk in the presence of some of the mutations (Park et al., 1997; Xieet al., 2004), there are many contradictory results apparently due to ethnic differences (Chatterjee et al., 2009; Zhou et al., 2009).

A transition from T to C in the 3’UTRof CYP1A1 gene results in the introduction of an MspI restriction site and is associated with increase in enzyme activity and hence cancer risk (Petersen et al., 1991;Tanimoto et al., 1999). In the present study we have observed that among the areca nut/pan masala chewers the risk of oral cancer and precancer been reduced with the TC genotype for T6235C polymorphism in the 3’UTRof CYP1A1 gene. C allele for T6235C polymorphism has been reported to increase the enzymatic activity of CYP1A1 enzyme.

The A4889G polymorphism in exon 7 of the CYPlAl gene results in an amino acid substitution from an isoleucine to a valine.22 This mutation occurs in the region of the gene which encodes the heme binding motif of the protein, and studies of benzo- [a] pyrene metabolism have shown that the valine protein demonstrates almost twice the enzyme activity of the isoleucine protein. Risk of many different cancers has been found to get altered with the A4889G polymorphism (Wu et al., 2013). The C4887A polymorphism is also located in exon 7, which exchanges threonine 461 with asparagine,and had shown association with the risk of non small cell lung cancer (Wright et al., 2010).

In conclusion our study suggests a possible association of polymorphisms in CYP1A1 gene with the risk of oral cancer and oral pre cancer. We documented that the both the genotypes and haplotypes of these polymorphisms modulate the risk of oral cancer and oral pre cancer. However, with the limited sample size our results constitute only a preliminary step in this direction and any clinical application is long way off.
